# Influence of Malnutrition According to the GLIM Criteria on the Clinical Outcomes of Hospitalized Patients With Cancer

**DOI:** 10.3389/fnut.2021.774636

**Published:** 2021-12-24

**Authors:** Chengyu Liu, Zhenhua Lu, Zijian Li, Jingyong Xu, Hongyuan Cui, Mingwei Zhu

**Affiliations:** ^1^Department of General Surgery, National Center of Gerontology, Beijing Hospital, Institute of Geriatric Medicine, Chinese Academy of Medical Sciences, Beijing, China; ^2^The Key Laboratory of Geriatrics, National Center of Gerontology, National Health Commission, Beijing Hospital, Beijing Institute of Geriatrics, Institute of Geriatric Medicine, Chinese Academy of Medical Sciences, Beijing, China; ^3^Department of Hepatopancreatobiliary Surgery, The Affiliated Hospital of Qinghai University, Xining, China; ^4^Department of Nutrition, National Center of Gerontology, Beijing Hospital, Institute of Geriatric Medicine, Chinese Academy of Medical Sciences, Beijing, China

**Keywords:** GLIM criteria, malnutrition, cancer patients, subjective global assessment, clinical outcome

## Abstract

**Background:** Malnutrition is prevalent among patients with cancer. The Global Leadership Initiative on Malnutrition (GLIM) released new universal criteria for diagnosing malnutrition in 2019. The objectives of this study were to assess the prevalence of malnutrition in patients with cancer using the GLIM criteria, explore the correlation between the GLIM criteria, and clinical outcomes, and compare the GLIM criteria with subjective global assessment (SGA).

**Methods:** This retrospective analysis was conducted on 2,388 patients with cancer enrolled in a multicenter study. Nutritional risk was screened using the Nutritional Risk Screening-2002, and the nutritional status was assessed using SGA and GLIM criteria. Chi-square analysis and Wilcoxon rank sum test, stratified by age 65 years, were used to evaluate the effect of GLIM-defined malnutrition on clinical outcomes. Logistic regression analysis was used to analyze the nutritional status and complications, and the interrater reliability was measured using a kappa test.

**Results:** The prevalence of malnutrition defined by the GLIM criteria was 38.9% (929/2,388). GLIM-defined malnutrition was significantly associated with in-hospital mortality (*P* = 0.001) and length of hospital stays (*P* = 0.001). Multivariate logistic regression analysis showed GLIM-defined malnutrition significantly increased complications (odds ratio [*OR*] 1.716, 95% *CI* 1.227–2.400, *P* = 0.002). The GLIM criteria had a “moderate agreement” (kappa = 0.426) compared with the SGA.

**Conclusions:** The prevalence of malnutrition in hospitalized patients with cancer is high, and malnourishment in patients with cancer is associated with poorer clinical outcomes. The use of the GLIM criteria in assessing the nutritional status of inpatients with cancer is recommended and can be used as the basis for nutritional interventions.

## Introduction

Malnutrition is a state resulting from a lack of intake or uptake of nutrition that leads to altered body composition (decreased fat-free mass) and body cell mass, which in turn lead to diminished physical and mental function and impaired clinical outcome from disease (1). Malnutrition is prevalent among patients with cancer in China and other countries (2–4). Malnutrition in patients with cancer is associated with incremental mortality and morbidity, extended length of hospital stays (LOS), and poorer quality of life (5–7). Studies have revealed that individualized nutritional support reduces the risk of mortality and improves the function and quality of life among the patients with cancer with higher nutritional risk, relative to when they were provided only with hospital nutrition (8). Nevertheless, the nutritional risk screening must be performed first from the time of cancer diagnosis, and patients with abnormal screening should undergo nutritional assessment before deciding whether to use nutritional therapy (9, 10).

The Global Leadership Initiative on Malnutrition (GLIM) developed and reported new diagnostic criteria for malnutrition in clinical settings (11). The GLIM criteria are based on expert opinion and require validation and reliability testing in different populations (11, 12). Currently, limited studies use the GLIM criteria to assess malnutrition in patients with cancer, and the methods are not uniform; some studies aimed to develop nomograms or scoring systems in combination with GLIM criteria to predict cancer survival (13, 14). Meanwhile, other studies have been performed in more specific cancer sites and populations, such as for esophageal tumors (15), hematological tumors (16), and outpatient with cancer (17). Therefore, the GLIM criteria still lack validation to diagnose malnutrition in hospitalized patients with cancer, and no study to date has comprehensively analyzed the inpatient clinical outcomes of patients with cancer using the GLIM criteria.

This study aimed to assess the prevalence of malnutrition in patients with cancer using the GLIM criteria, explore the correlation between the GLIM criteria, and clinical outcomes, such as complication rate, and compare the GLIM criteria with subjective global assessment (SGA).

## Methods

### Study Subjects

This retrospective analysis was conducted on a prospective observational study initiated at 34 large hospitals in 18 cities in China from June to September 2014. Inclusion criteria were inpatients with cancer over 18 years old, patients who were conscious, and patients who signed the informed consent form. The exclusion criteria were inpatients with cancer who had been hospitalized for <7 days or more than 30 days and patients who underwent surgery before 8:00 the next day. The study protocol was approved by the Ethics Committee of Beijing Hospital (PIC approval number: 2014BJYYEC-022-02) and registered in the Chinese Clinical Trial Registry (Registered No. ChiCTR-EPC-14005253).

### Data Collection

Anthropometric measurements, such as height, weight, arm circumference, calf circumference, and handgrip strength were measured using standard measurement methods within 24 h of admission (18). Laboratory test results included blood proteins (total protein, albumin, and prealbumin) and blood lipids (cholesterol and triglycerides). The primary outcome was the incidence of complications. Complications were defined as any deviation from the ideal course of treatment during hospitalization, excluding failure to cure. Secondary outcomes were in-hospital death, infectious complications, intensive care unit (ICU) admission, LOS, total hospital cost, and LOS in the ICU. Infectious complications were defined as the presence of a pathogen in an otherwise sterile tissue and confirmed by its culture, or the presence of clinical symptoms and signs and radiological or hematological evidence associated with the infection. The remaining clinical outcomes were recorded by searching the medical records system.

### Nutritional Screening and Assessment

The prevalence of nutritional risk and malnutrition among the inpatients with cancer was prospectively defined using nutritional risk creening-2002 (NRS-2002) and SGA, and retrospectively defined using the GLIM criteria.

#### GLIM Criteria

The GLIM criteria are a two-step model for risk screening and diagnosis. The first step is to screen out patients at nutritional risk using the NRS2002 and the second step is to assess for the malnutrition diagnosis and severity grading in patients at nutritional risk. The NRS-2002 is graded and scored according to the nutritional status, disease severity, and age, and a score of 3 or greater was considered nutritional risk (19). The second step of GLIM criteria are composed of phenotypic criteria (non-volitional weight loss, low BMI, and reduced muscle mass) and etiologic criteria (reduced food intake or assimilation and disease burden/inflammation), the diagnosis of malnutrition requires at least phenotypic criterion and etiological criterion (11).

As cancer meets the etiological criterion of GLIM criteria, patients who meet one of the three phenotypic criteria are diagnosed with malnutrition. Weight loss >5% within the past 6 months or >10% beyond 6 months was judged to be non-volitional weight loss. Low BMI using the Asian BMI data (18.5 and 20 kg/m^2^ for patients aged <70 years and ≥ 70 years, respectively). There were no results of body composition analysis of muscle mass in this study; hence, calf circumference and handgrip strength were used as alternative measures (11). According to the Asian Working Group for Sarcopenia 2019 Consensus (20), those with calf circumference <34 cm (male) or <33 cm (female) had low calf circumference, those with handgrip strength <28 kg (male) or <18 kg (female) had reduced handgrip strength, and patients with both low calf circumference and low handgrip strength were judged to have reduced muscle mass in this study. Having one of two conditions of (i) weight loss >10% within the past 6 months or >20% beyond 6 months and (ii) BMI <17.0 kg/m^2^ if age <70 years or <17.8 kg/m^2^ if age ≥ 70 years was diagnosed as Stage 2 (severe malnutrition) (21). Stage 1 (moderate malnutrition) was diagnosed as a malnutrition without meeting severe malnutrition conditions. Serum albumin can be used as a supportive proxy measure of inflammation, and those with serum albumin concentrations <35 g/L were defined as having hypoalbuminemia (11, 22, 23).

#### Subjective Global Assessment

Patients were divided into three levels by assessing eight indicators: weight change, dietary intake changes, gastrointestinal symptoms (that persisted >2 weeks), functional capacity, disease and its relation to nutritional requirements, loss of subcutaneous fat, muscle wasting, and ankle edema in the past 2 weeks (24, 25). These eight aspects are classified into grades A, B, and C. At least five items belong to grades B or C and can be rated as moderate or severe malnutrition, respectively.

### Statistical Analysis

Continuous variables were expressed as mean (SD) or median (interquartile range [IQR]), and differences were analyzed by *t*-test or rank sum test. Categorical variables were expressed as frequencies (percentages), and differences were determined by the chi-square test. Chi-square analysis and rank sum test, stratified by age at 65 years, were used to evaluate the effect of GLIM-defined malnutrition on the incidence of complications, infectious complications, and other clinical outcomes. Univariate logistic regression analysis was used to investigate the association between demographic characteristics, nutritional status, and GLIM criteria and the occurrence of complications, and multivariate logistic regression analysis (forward: likelihood ratio) adjusted for age and gender was used to analyze the association between GLIM-defined malnutrition and the occurrence of complications. Patients with SGA grades B and C were considered malnourished for the analysis. The kappa test was used to investigate the rate of agreement between the GLIM criteria and SGA, and Spearman's rank correlation analysis was performed. A kappa value of 0.21–0.40 was considered as fair agreement; 0.41–0.60 as moderate agreement; and 0.61–0.80 as substantial agreement (26). The GLIM criteria were compared by receiver operating characteristic (ROC) analysis using SGA as a reference tool, and the area under the curve (AUC) was calculated. SPSS 25 version was used for statistical analysis, and a *P* <0.05 was considered statistically significant.

## Results

### Study Characteristics

The demographic and clinical characteristics of the 2,388 cancer patients are shown in Table 1. In this study, such as 1,523 men and 865 women, 854 of whom were elderly (≥ 65 years old) and 1,534 were younger than 65 years old. There were 576 patients with colorectal cancer, 563 patients with gastric cancer, and 544 patients with lung cancer.

**Table 1:** Demographic and clinical characteristics of the patients with cancer.

**Characteristic**	**Total**	**With malnutrition**	**Without malnutrition**	**P-value**
	**(*n =* 2,388)**	**(*n =* 929)**	**(*n =* 1,459)**	
Gender male (%)	1,523 (63.8%)	608 (65.4%)	915 (62.7%)	0.176
≥65y, n (%)	854 (34.3%)	438 (51.2%)	416 (28.5%)	<0.001
Height (cm)	166.13 ± 7.77	165.69 ± 7.65	166.40 ± 7.84	0.030
Body weight (kg)	62.69 ± 11.27	57.48 ± 10.93	66.01 ± 10.17	<0.001
BMI (kg*m^−2^)	22.67 ± 3.46	20.89 ± 3.47	23.80 ± 2.95	<0.001
Leukocyte count (10^9*^L^−1^)	2.74 ± 5.75	2.68 ± 5.63	2.77 ± 5.82	0.701
Total protein (g*L^−1^)	66.35 ± 6.52	65.14 ± 6.85	67.11 ± 6.18	<0.001
Albumin (g*L^−1^)	39.29 ± 5.03	37.81 ± 5.17	40.22 ± 4.71	<0.001
Prealbumin (g*L^−1^)	0.24 ± 0.08	0.22 ± 0.08	0.25 ± 0.07	<0.001
TG (mmol*L^−1^)	1.61 ± 1.33	1.45 ± 1.33	1.70 ± 1.33	0.001
TC (mmol*L^−1^)	4.29 ± 1.29	4.19 ± 1.25	4.35 ± 1.31	0.021

*All values are expressed as mean ± SD or number of patients (%)*.

*BMI, body mass index; TG, total triglyceride; TC, total cholesterol*.

### Prevalence of Malnutrition in Patients With Cancer at Admission

The prevalence of malnutrition based on GLIM criteria at admission in patients with cancer was 38.9% (929/2,388), including 27.0% (644/2,388) with moderate malnutrition and 11.9% (285/2,388) with severe malnutrition. There were 1,212 (50.8%) patients at nutritional risk screened by NRS-2002. In total, 663 (27.8%) patients had non-volitional weight loss, 318 (13.3%) patients had low BMI, and 283 (11.9%) patients had reduced muscle mass. Among the three cancer types, the prevalence of nutritional risk and malnutrition was the highest (49.4%) in patients with gastric cancer and the lowest (25.7%) in patients with lung cancer (Table 2).

**Table 2:** The nutritional status of patients with cancer grouped by disease type.

		**Nutritional risk**		**Malnutrition (SGA B or C)**		**Malnutrition (GLIM criteria)**	
	**N**	**N**	**Percent**	**N**	**Percent**	**N**	**Percent**
All	2,388	1,212	50.8%	777	32.5%	929	38.9%
Colorectal cancer	576	308	53.5%	198	34.4%	231	40.1%
gastric cancer	563	369	65.5%	217	38.5%	278	49.4%
lung cancer	544	197	36.2%	118	21.7%	140	25.7%

*SGA, subjective global assessment; GLIM, Global Leadership Initiative on Malnutrition; N, number*.

### Correlation Between GLIM-Defined Malnutrition and Clinical Outcomes

In-hospital mortality for all patients was 0.5% (11/2,388); 10 patients were malnourished, and only one was not. The overall complication rate was 6.1% (146/2,387), with 8.1% (75/929) in patients with malnutrition, and 4.9% (71/1,458) in patients without malnutrition.

Malnutrition was significantly associated with the incidence of death (1.1 vs. 0.1%, *P*_Fisher_ = 0.001), complications (8.1 vs. 4.9%, χ^2^ = 10.141, *P* = 0.001), infectious complications (5.0 vs. 3.2%, χ^2^ = 4.942, *P* = 0.026), ICU admission (7.3 vs. 5.1%, χ^2^ = 4.769, *P* = 0.029), LOS (*P* = 0.001), and LOS in the ICU (*P* = 0.027). The LOS and LOS in the ICU of patients with malnourished cancer were significantly longer than those of patients without malnutrition (*P* = 0.001; *P* = 0.027), but there was no significant difference in the total hospital cost (*P* = 0.746).

After stratification by age at 65 years, malnutrition was significantly associated with the occurrence of death and complications in non-elderly and elderly patients (Table 3).

**Table 3:** The association between GLIM defined malnutrition and clinical outcomes.

	**≥** **65 years**, ***n*** **=** **854**			**<** **65 years**, ***n*** **=** **1,534**		
	**With malnutrition**,	**Without malnutrition**,	***P*-value**	**With malnutrition**,	**Without malnutrition**,	***P*-value**
	***n* = 438**	***n* = 416**		***n* = 491**	*n* =1,044^*^	
Mortality	6 (13.7%)	0 (0%)	0.031	4 (0.8%)	1 (0.1%)	0.039
Complications	37 (8.45%)	19 (4.57%)	0.022	38 (7.74%)	52 (4.99%)	0.033
Infectious complications	23 (5.25%)	10 (2.40%)	0.031	23 (4.68%)	36 (3.45)	0.243
ICU admission	37 (8.45%)	24 (5.77%)	0.129	31 (6.31%)	51 (4.89%)	0.249
LOS, days	14.00 (11.00)	13.00 (10.00)	0.224	13.00 (11.00)	12.00 (9.00)	0.028
Total hospital cost, USD	4,447.67 (6,495.54)	4,661.05 (6,622.70)	0.604	4,143.51 (6,703.89)	4,224.63 (6,129.00)	0.789
LOS in the ICU, days	0.00 (0.00)	0.00 (0.00)	0.131	0.00 (0.00)	0.00 (0.00)	0.232

*All values are expressed as median (IQR), or n (%)*.

* *1 non-malnourished patient under 65 years had only a LOS, and the remaining clinical outcome records were missing*.

*GLIM, Global Leadership Initiative on Malnutrition; LOS, length of stay; ICU, intensive care unit; USD, United State dollar; IQR, interquartile range*.

### Correlation Between GLIM Criteria and Complications

**Figure 1:**
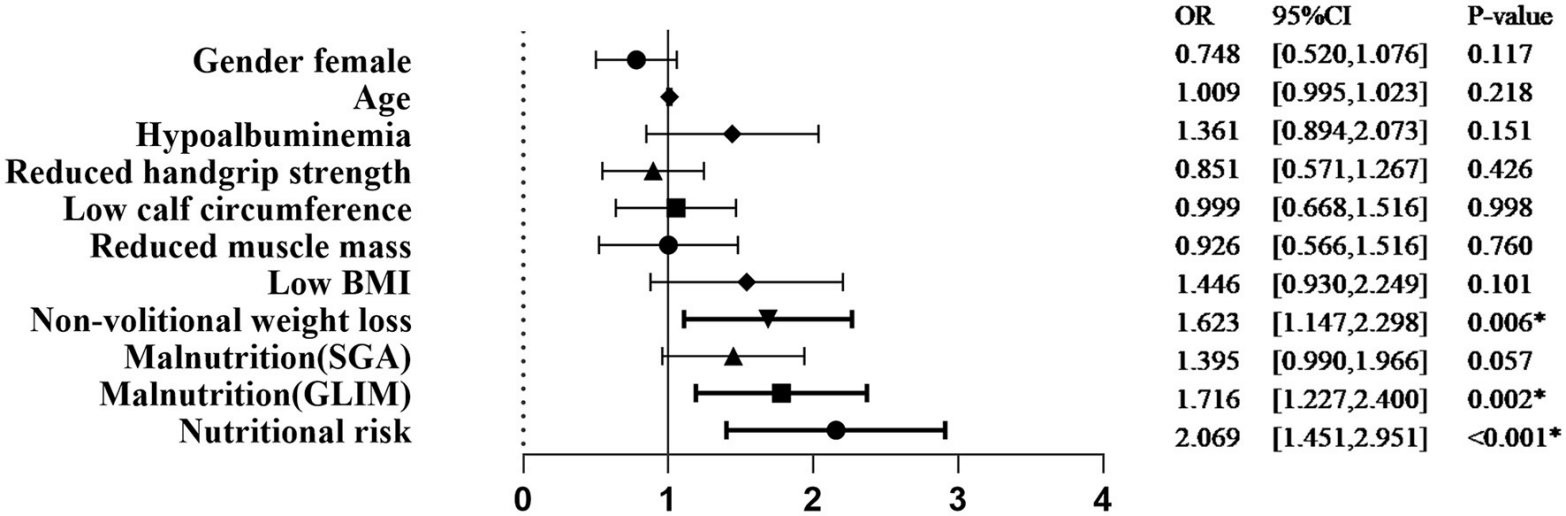
Univariate analysis of demographic characteristics, nutritional status, and GLIM criteria associated with complications. SGA, subjective global assessment; GLIM, Global Leadership Initiative on Malnutrition; BMI, body mass index; OR, odds ratio. *p < 0.05.

Univariate logistic regression analysis showed that the nutritional risk, GLIM-defined malnutrition, and non-volitional weight loss were associated with significantly increased complications (Figure 1).

The association of GLIM-defined malnutrition with the occurrence of complications was maintained when age and gender were added as covariates in multivariate logistic regression analysis (odds ratio [*OR*] 1.716, 95% CI 1.227–2.400, *P* = 0.002).

### Comparison Between GLIM Criteria and SGA

**Figure 2:**
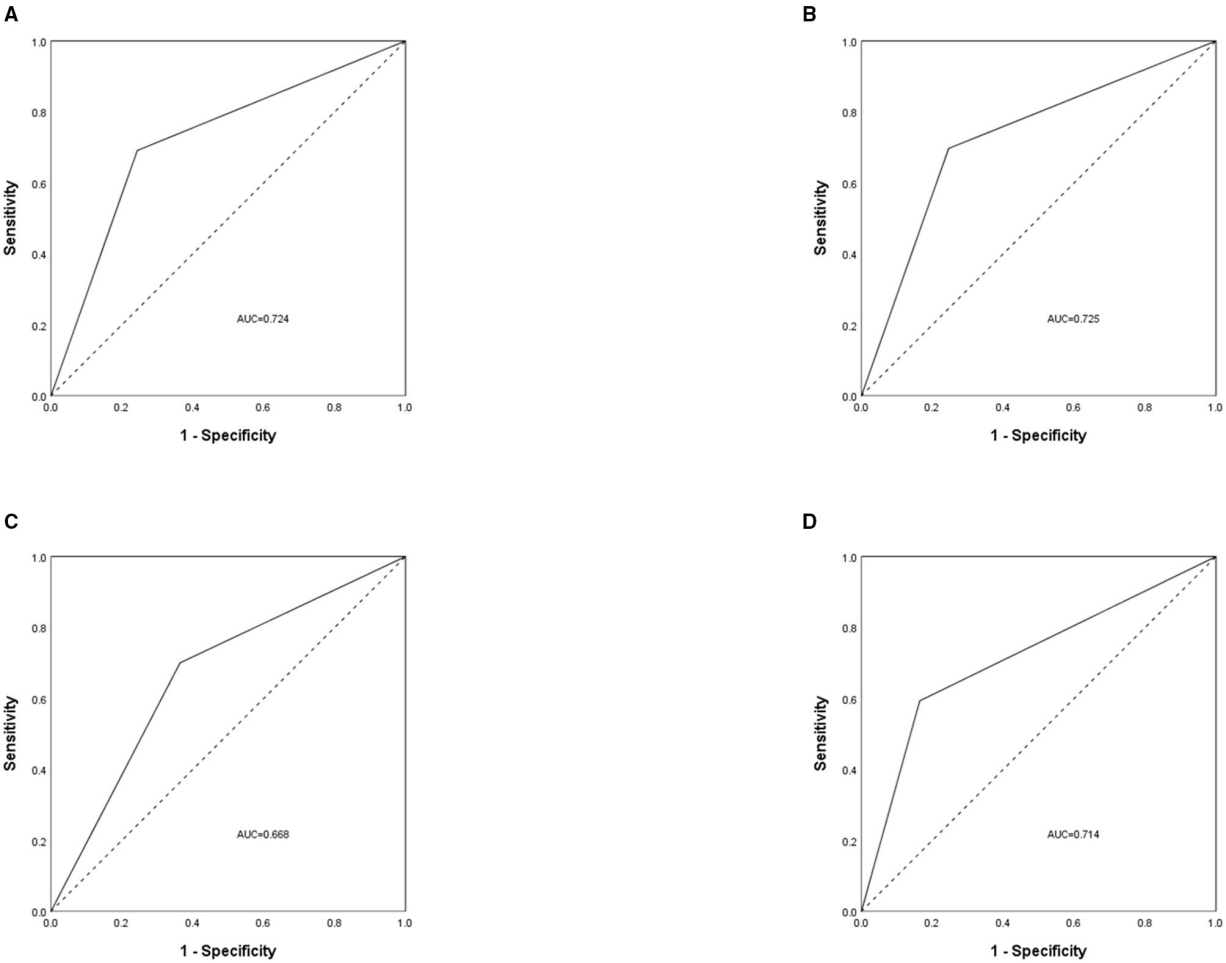
The receiver operating characteristic (ROC) curve was used to assess the difference between patients with malnutrition and without malnutrition, measured by Global Leadership Initiative on Malnutrition (GLIM) criteria and subjective global assessment (SGA). The analysis was grouped by disease type, with **(A)** for all cancer patients, **(B)** for patients with colorectal cancer, **(C)** for patients with gastric cancer, and **(D)** for patients with lung cancer. AUC, area under the curve.

The prevalence of malnutrition according to the SGA category was 32.5% (777/2,388), with 28.9% (689/2,388) and 3.7% (88/2,388) in SGA grades B and C, respectively. Table 4 showed the proportion of patients assessed as malnourished by SGA and GLIM criteria. The GLIM criteria had a sensitivity of 69.1% and a specificity of 24.3% (kappa = 0.426, moderate agreement) when compared with SGA (Spearman, r_s_ = 0.430, *P* < 0.001), and ROC analysis showed that the AUC of the GLIM criteria was 0.724 (*P* < 0.001). The ROC curve analyzed according to disease type is shown in Figure 2.

**Table 4:** Number of patients with cancer identified as malnourished by SGA and GLIM.

	**SGA B or C**		
GLIM Malnourished	No	Yes	Total n (%)
No	1,219	240	1,459 (61.1%)
Yes	392	537	929 (38.9%)
Total n (%)	1,611 (67.5%)	777 (32.5%)	2,388

*SGA, subjective global assessment; GLIM, Global Leadership Initiative on Malnutrition*.

## Discussion

Our study found that the prevalence of patients with cancer diagnosed according to the GLIM criteria for malnutrition was 38.9%, which resulted in poorer clinical outcomes, such as a higher incidence of complications and longer LOS. And there was moderate consistency between the GLIM criteria and SGA among patients with cancer.

Malnutrition is prevalent in patients with cancer. Marshall et al. (4) found that 26–31% of patients with cancer in Australia were malnourished. Zhang et al. (27) observed that 28.3% of patients with cancer in eastern China were malnutrition at admission according to the GLIM criteria. Zhang et al. (28) found that 48.4% of elderly patients with cancer in China were diagnosed with malnutrition. The prevalence of malnutrition in elderly patients with cancer is significantly higher than that in adult patients. Frailty, sarcopenia, and functional impairment are important risk factors for malnutrition in elderly patients with cancer (29). Elderly malnourished patients with cancer have a higher risk of death and prolonged LOS (13, 28, 30).

Metabolic changes in patients with cancer are mainly alterations in the ability to utilize nutrients, which are caused by tumors or cancer therapy, producing chronic inflammation and excess catabolism (31, 32). Therefore, cancer is associated with chronic or recurrent inflammation that meets the etiologic criterion of disease burden/inflammation of the GLIM criteria (11).

The mechanisms by which malnutrition affects clinical outcomes in patients with cancer are as follows: first, patients with cancer have reduced food intake due to systemic effects of the tumor and have altered nutritional metabolism and resting energy expenditure (33). Second, malnutrition affects the function and recovery of every organ system (34). Third, malnutrition may lead to some adverse events, such as weakened immune response, reduced tolerance to chemotherapy/immunotherapy, negative treatment outcomes, increased infection rates, increased risk of postoperative complications, and thus reduced quality of life (13, 32, 35–37).

The applicability of the GLIM criteria in patients with cancer has been validated in some studies. Zhang et al. (29) retrospectively developed a nomogram combining the GLIM criteria with other variables and found that the GLIM criteria could be used to predict the 1-year and 2-year overall survival rates. Gascón-Ruiz et al. (38) prospectively showed that the prevalence of malnutrition was higher using the GLIM criteria than using the ESPEN criteria in outpatients with cancer, and the GLIM criteria were helpful for early intervention in patients with cancer. Some studies have also pointed out that the GLIM criteria are significantly associated with the risk of death in lung cancer, hematological tumors, etc. (16, 39).

Validation of the GLIM criteria requires comparison with other nutritional assessment tools. SGA is a validated and reliable tool for nutritional assessment in patients with cancer (24, 40–42). GLIM criteria had a “moderate agreement” (kappa = 0.426) compared with SGA. The difference in prevalence rates between SGA and GLIM criteria can be explained by the differences in the assessment criteria for BMI and muscle mass. Allard et al. (43) compared GLIM criteria with SGA, and the results showed that the weight loss with either high C-reactive protein (CRP) or low intake had high specificity but very low sensitivity for the diagnosis of malnutrition. Balci et al. (44) retrospectively found a good consistency between GLIM criteria and SGA in patients hospitalized for acute illnesses (*k* = 0.804).

There are some limitations. First, since our study was a retrospective study, many covariables were missing from our medical record system and were not included in our final analysis but can be collected in future prospective studies. Second, for the assessment of reduced muscle mass, we selected handgrip strength combined with calf circumference without the use of the recommended appendicular skeletal muscle index, which introduced a bias. However, this is inevitable in studies that lack dual energy X-ray absorptiometry or bioelectrical impedance analysis to measure muscle mass. Third, due to the limited sample size, our analysis using stratification at 65 years may not have sufficient ability to detect correlations. Fourth, our study is based on the Chinese patients with cancer, and the findings are not representative of patients with cancer in other ethnic groups.

Patients with cancer should be regularly screened for the risk of malnutrition or for malnutrition from the time of diagnosis of the tumor, and the GLIM criteria are recommended, followed by timely standardized nutritional interventions for malnourished patients with cancer (9, 10). Future studies need to prospectively verify the efficacy of the GLIM criteria and explore the combination of GLIM criteria used to predict different outcomes in patients with cancer (11, 12).

In conclusion, this study showed a high prevalence of malnutrition in patients with cancer; malnourished patients with cancer had poorer clinical outcomes, such as a higher incidence of complications and longer LOS. The use of the GLIM criteria is a standardized method of assessing the nutritional status of patients with cancer and can be used as a framework for subsequent nutritional treatment interventions, such as personalized nutritional therapy.

## Data Availability Statement

The raw data supporting the conclusions of this article will be made available by the authors, without undue reservation.

## Ethics Statement

The studies involving human participants were reviewed and approved by Ethics Committee of Beijing Hospital. Written informed consent to participate in this study was provided by the participants' legal guardian/next of kin.

## Author Contributions

CL and MZ designed the research and methodology. ZLi and JX performed data management. CL analyzed the data. CL and ZLu drafted the manuscript. HC and MZ critically reviewed the manuscript. All authors agreed the final version of the manuscript.

## Funding

Food Science Foundation of Chinese Institute of Food Science and Technology (2020-14) and Transformation Project of Scientific and Technological Achievements in Qinghai Province(2020-SF-162).

## Conflict of Interest

The authors declare that the research was conducted in the absence of any commercial or financial relationships that could be construed as a potential conflict of interest.

## Publisher's Note

All claims expressed in this article are solely those of the authors and do not necessarily represent those of their affiliated organizations, or those of the publisher, the editors and the reviewers. Any product that may be evaluated in this article, or claim that may be made by its manufacturer, is not guaranteed or endorsed by the publisher.
